# Off-season seed multiplication in spring barley: a scale-up step for speed breeding

**DOI:** 10.3389/fpls.2026.1912336

**Published:** 2026-08-20

**Authors:** Alina G. Marenkova, Vladimir N. Igonin, Alina A. Kocheshkova, Kroupin P. Yu, Mikhail G. Divashuk

**Affiliations:** Department of Applied Genetic Technologies, All-Russia Research Institute of Agricultural Biotechnology, Moscow, Russia

**Keywords:** breeding pipeline, controlled environment, high-pressure sodium, *Hordeum vulgare*, LED, off-season multiplication, seed multiplication ratio (SMR), speed breeding

## Abstract

Speed breeding (SB) accelerates generation turnover, but the small seed quantities produced during rapid generation advance remain a bottleneck. Here, we quantify intensive off-season seed multiplication as a scale-up stage of the SB pipeline. Using spring barley, we tested two lighting systems, high-pressure sodium (HP S) and light-emitting diode (LED), and two cultivars at three pot configurations in Moscow (55.75°N) over winter at 24°C and a 22-h photoperiod. Fertile tillers reached 31–105 per plant, and the mean seed number ranged from 553 to 1,562 per plant, giving a seed multiplication ratio ( SMR; seeds harvested per seed sown) of up to ~1,560, against typical field values of ~30–120 seeds per plant. The response was reproduced in both cultivars. Per lamp-supplied photon, HP S matched or exceeded LED efficiency in all three pot configurations, indicating that the higher LED yields reflect a greater photon dose rather than spectral superiority. Per unit area, 2 L pots were the most efficient (~20,000–24,500 seeds m^−2^), so a single winter cycle in ~1.3–3.3 m^2^ of chamber space can supply enough seed for a competitive yield trial and save up to two breeding years. This is a proof of concept based on two cultivars, one winter season, one site, and two replicates; within those limits, the approach requires no lighting beyond that already installed in controlled-environment facilities.

## 1 Introduction

Barley (*Hordeum vulgare* L.) is a globally important cereal and the main raw material for malting. It is a long-day species that responds strongly to an extended photoperiod, which makes it particularly amenable to speed breeding (SB): protocols are now available that deliver several generations per year (Watson et al., 2018; Ghosh et al., 2018), and we have previously established and refined such a protocol for malting barley (Marenkova et al., 2024). The field has been reviewed recently (Blinkov et al., 2025; Kabade et al., 2025). Even so, the development of a malting cultivar remains slow, because faster generation turnover is only one of the steps that determine how quickly a line reaches replicated field testing.

Existing SB protocols are optimized for generation turnover: immature ears are harvested, and single-seed descent is used, so each cycle yields only a minimal quantity of seed by construction. Yield testing, however, progresses through a breeding nursery, a control nursery, and preliminary and competitive variety trials, each requiring more seed than the last. A competitive variety trial entry for spring barley may require 30,000–80,000 seeds, while earlier stages need proportionally fewer. If a selected line cannot be multiplied quickly enough, the time gained by rapid generation advance is lost again during field multiplication, and this scale-up step has received little quantitative attention.

Off-season multiplication in a controlled environment can bridge this gap: a selected genotype is grown to full maturity over winter, and the bulked seed is sown into replicated trials the following spring, bypassing one or two field seasons.

Moreover, SB facilities differ in lighting infrastructure—high-pressure sodium (HP S) lamps, emitting mainly yellow–orange with little blue light, or spectrally tunable light-emitting diodes (LEDs) (Ouzounis et al., 2015). The original glasshouse SB protocol used HP S at ~440–650 µmol m^−2^ s^−1^ (Watson et al., 2018), whereas later chamber protocols employ LEDs (Ghosh et al., 2018; Jähne et al., 2020). A direct comparison of the two systems for intensive multiplication of spring barley is lacking, and where the systems also differ in photon flux, claims of spectral superiority can be misleading.

Here, we ask whether intensive off-season multiplication can serve as a practical scale-up step under either of the two lighting systems in common use. We evaluated two spring barley cultivars at three pot configurations under HP S and LED lighting, measuring the seed multiplication ratio (SMR), lamp-light use efficiency (L-LUE) and area-based yield. We (1) quantify the seed output attainable in one winter cycle, (2) test whether comparable output is obtained under both lighting systems, and (3) give practical guidance on pot choice.

## 2 Materials and methods

### 2.1 Plant material and growth conditions

Two two-rowed malting spring barley cultivars, Grace and Fatima (**Supplementary Table S1**), were sown on 30 December 2025 in Moscow (55.75°N) without pre-germination, two replicates per cultivar. All treatments received 24°C, a 22-h photoperiod, and 41.5% relative humidity (RH) (Marenkova et al., 2024).

Two lighting configurations were compared. The HP S treatment used high-pressure sodium lamps providing a photosynthetic photon flux density (PPFD, 400–700 nm) of 470 µmol m^−2^ s^−1^ at ear height. This value lies within the range of 440–650 µmol m^−2^ s^−1^ used in the original glasshouse speed-breeding protocol (Watson et al., 2018). The LED treatment used broad-spectrum LED panels (blue peak, ~450–680 nm) at 858 µmol m^−2^ s^−1^, housed in a glasshouse with supplementary daylight, whereas the HP S system received no daylight. Spectra were measured with a portable spectroradiometer UPRtek PG200N Spectral PAR Meter (UPRtek, Miaoli County, Zhunan Township, Taiwan). The lamp-only daily light integral (DLI) was ≈37 mol m^−2^ day^−1^ (HP S) and ≈68 mol m^−2^ day^−1^ (LED); the LED treatment additionally received an estimated 1–3 mol m^−2^ day^−1^ of PAR from daylight. Thus, the systems differed simultaneously in spectrum, photon flux, and daylight contribution; they are treated as integral technological alternatives. Details of irrigation, pest control, fertilizer application, and statistical analysis are given in the **Supplementary Methods**.

### 2.2 Measurements and data analysis

Fertile and total tillers, seed number, seed mass, thousand-grain weight (TGW), and days to maturity were recorded for every pot. The design was a balanced factorial: 2 lighting systems × 3 pot configurations × 2 cultivars × 2 replicates (24 pots). All 24 pots were retained in the analysis, so the design remained fully balanced and the results are independent of the type of sums of squares ( S S) used. A three-way analysis of variance was performed with effect sizes reported as *η*². The model included lighting, pot, cultivar, and lighting × pot; no interaction involving cultivar approached significance (*p* ≥ 0.09), and all were dropped, giving residual *df* = 17. Planned contrasts separated pot volume (10 L versus 2 L, one plant) and planting density (one versus three plants per 2 L pot). Residual diagnostics ( Shapiro–Wilk test and visual inspection) indicated no severe violations of the ANOVA assumptions. One pot (HP S, 2 L with three plants, cv. Grace, replicate 1) had a TGW of 27.3 g, identified as an outlier by Grubbs’ test (*p* < 0.05) and attributable to incomplete grain filling in an early-harvested sample; it was retained in the main analysis, and a sensitivity analysis excluding it is given in **Supplementary Table S3**. Both analyses lead to the same conclusions. Full ANOVA results are given in **Table 1**; **Supplementary Table S2**.

**Table 1:** Three-way analysis of variance for fertile tillering, seed number, seed mass, and thousand-grain weight (TGW).

Source of variation	Fertile tillers	Seed number	Seed mass	TGW
Lighting	63.5*** (35.8)	23.3*** (14.4)	9.1** (9.8)	0.7 ns (2.4)
Pot configuration	35.3*** (39.8)	48.3*** (59.8)	27.6*** (59.4)	1.7 ns (11.9)
Cultivar	0.03 ns (0.0)	0.1 ns (0.1)	0.9 ns (0.9)	7.3* (25.0)
Lighting × pot	13.1*** (14.8)	12.3*** (15.2)	5.3* (11.5)	0.4 ns (2.8)
Residual	– (9.6)	– (10.5)	– (18.3)	– (57.9)

*F* values are given with their significance, and effect sizes (*η*², % of total variation) in parentheses. All 24 pots were included; residual *df* = 17. Interactions involving cultivar did not approach significance (*p* ≥ 0.09) and were dropped from the model.

***p < 0.001; **p < 0.01; *p < 0.05; ns, not significant.

Because each factor level is represented by 8 to 12 pots, the sensitivity of the tests is higher than the two-fold replication of an individual cell suggests. With a residual standard deviation of 144 seeds per pot, the minimum detectable difference at 80% power and *α* = 0.05 was 175 seeds for a main effect, 214 seeds between levels of the pot factor, and 303 seeds between two individual treatments. We report these values together with 95% confidence intervals rather than retrospective power computed from the observed effect, which is a monotone function of the achieved *p*-value and carries no independent information. Non-significant effects are accordingly interpreted as differences that were not detected rather than as differences shown to be absent. Analyses were performed in Python 3.12.3 using statsmodels (v. 0.14.6) and SciPy (v. 1.17.1).

L-LUE was calculated for each pot as seeds (or grams) per mole of lamp-supplied photons (DLI × days to maturity × pot footprint), using DLI values of 37.2 mol m^−2^ day^−1^ (HP S) and 68.0 mol m^−2^ day^−1^ (LED), and is reported as the mean of the per-pot values. These are upper-bound estimates, since they assume full interception of light within the projected area of the pot. Per-pot raw data for all 24 experimental units are given in **Supplementary Table S5**.

## 3 Results

### 3.1 Growing period and tillering

The growing period was 73–105 days (mean ~91) under HP S and 92–105 days (mean ~100) under LED; heading under HP S was approximately 10 days earlier. These durations overlap with field values, 70–92 days (State Commission of the Russian Federation for Selection Achievements Test and Protection, 2026), confirming that the benefit lies in off-season timing rather than phenological acceleration.

**Figure 1:**
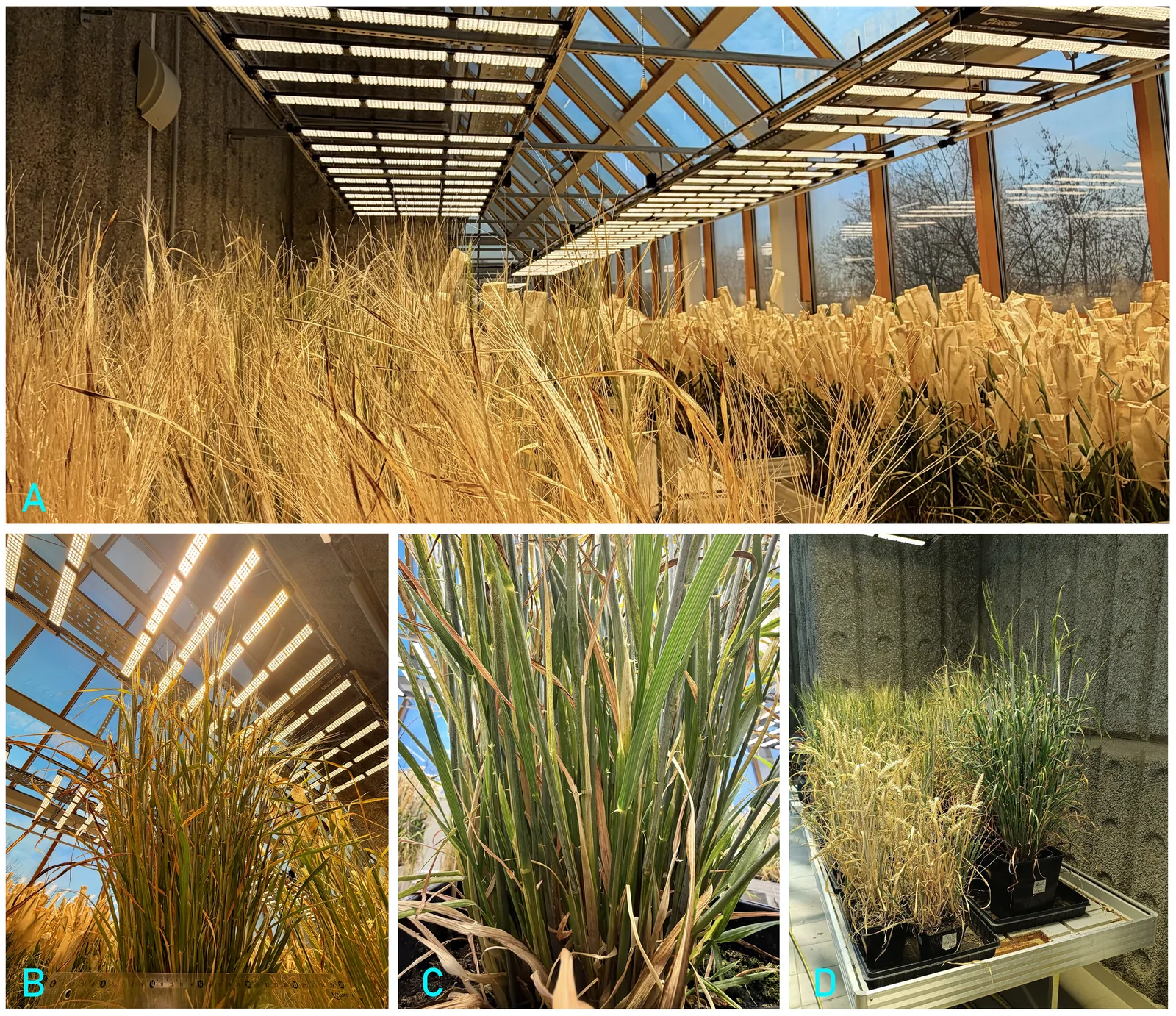
Spring barley under speed-breeding conditions. **(A)** General view of the mature stand under LED panels. **(B)** A single plant showing intensive fertile tillering. **(C)** Close-up of the tiller base. **(D)** Comparison of a small (2 L) and a large (10 L) pot.

Fertile tiller number reached 31–105 per plant across treatments (**Figure 1**), vastly exceeding typical field values of 1.5–3.2 (Abeledo et al., 2004). Pot configuration (*η*² ≈ 40%) and lighting (*η*² ≈ 36%) both significantly affected tillering (p < 0.001), as did the lighting × pot interaction (*η*² ≈ 15%; **Table 1**). For seed number and mass, pot configuration explained approximately 60% of the variation, and no effect of cultivar was detected (*η*² < 1%). TGW was affected only by cultivar (*η*² = 25.0%, *p* = 0.015), with Fatima forming heavier grain; no other factor had a detectable effect on TGW.

### 3.2 Seed multiplication and pot configuration

Mean seed number per plant ranged from 553 (HP S, 2 L, one plant) to 1,562 (LED, 10 L, one plant), giving an SMR, defined as the number of seeds harvested per seed sown, of ~550–1,560 (**Table 2**). For comparison, a field-grown barley plant sets the order of 30–120 seeds. The highest per-plant output was obtained in 10 L pots under LED: 105 tillers, 1,562 seeds, 70 g (**Table 2**). Individual replicates in this treatment reached up to 125 tillers and 1,819 seeds. However, per unit area, 2 L pots were most efficient (~20,000–24,500 seeds m^−2^). Increasing density from one to three plants per 2 L pot did not increase per-pot output (*p* ≥ 0.39); individual plants yielded ~200 seeds, dropping the SMR per sown seed to ~200. For comparison, a field crop yielding 3–5 t ha^−1^ with a TGW of approximately 45 g produces approximately 6,700–11,100 seeds m^−2^, so the chamber exceeded field seed output per unit area by roughly two- to fourfold. Expressed per liter of substrate, the smaller pot was also the more efficient (277–343 seeds per liter versus 87–156 in the 10 L pot), which indicates that the response is not proportional to the amount of substrate and fertilizer supplied but shows signs of saturation. Thus, one plant per pot is preferable for multiplication. All main effects were reproduced in both cultivars (**Table 3**), although with only two cultivars this indicates consistency within the material tested rather than independence of genotype. The three key contrasts were LED versus HP S in 10 L pots (difference 691 seeds; 95% CI 476 to 905), 10 versus 2 L under LED (876 seeds; 662 to 1,091), and 10 versus 2 L under HP S (318 seeds; 103 to 532); the last of these lies close to the resolution limit of the design (minimum detectable difference 303 seeds) and is therefore treated with caution. The main effect of lighting on seed number was 284 seeds, whereas the difference between cultivars was 19 seeds (798 versus 818), well below the 175 seeds the design could have detected. The distribution of seed number per plant across treatments is shown in **Supplementary Figure S1**.

**Table 2:** Mean productivity, seed multiplication ratio ( SMR), and area-adjusted seed yield per treatment (pooled over cultivars; *n* = 4).

System	Pot volume, L/plants in pot, pcs	Fertile tillers	Seed number	SMR	Seeds m^−2^
HP S	10 L/1	45.5 (36–50)	871 (748–955)	871	9,400
HP S	2 L/1	31.3 (26–38)	553 (443–658)	553	19,800
HP S	2 L/3*	32.2* (29–36)	574.5* (519–601)	192	20,500
LED	10 L/1	104.8 (87–125)	1,562 (1,273–1,819)	1,562	16,800
LED	2 L/1	51.8 (45–59)	685 (515–856)	685	24,500
LED	2 L/3*	46.0* (39–53)	603* (512–776)	201	21,500

Min–max in parentheses. For “2 L + 3 plants,” fertile tillers and seed number are per pot (three plants); SMR is per sown seed. All 24 pots are included. For the HP S 2 L/3 treatment, the mean TGW was 41.0 g (45.6 g if the early-harvested outlier is excluded), and the mean seed mass was 23.5 g (25.9 g); see **Supplementary Table S3**.

*Per pot (three plants); SMR per sown seed.

**Table 3:** Per-cultivar means for each treatment (*n* = 2).

System	Pot	Cultivar	Fertile tillers	Seeds/plant	Mass (g)	TGW (g)
HP S	10 L	Grace	48.0 (46–50)	920	43.5	47.3
HP S	10 L	Fatima	43.0 (36–50)	822	39.0	47.5
HP S	2 L	Grace	27.0 (26–28)	482	19.8	41.2
HP S	2 L	Fatima	35.5 (33–38)	625	31.7	50.7
LED	10 L	Grace	106.0 (87–125)	1,638	76.1	45.7
LED	10 L	Fatima	103.5 (92–115)	1,485	64.2	43.5
LED	2 L	Grace	51.5 (46–57)	654	25.6	39.8
LED	2 L	Fatima	52.0 (45–59)	717	32.6	45.8
HP S	2 L/3	Grace	30.5 (29–32)	558.5	19.1	34.87
HP S	2 L/3	Fatima	34.0 (32–36)	590.5	27.9	47.13
LED	2 L/3	Grace	46.5 (40–53)	538.5	20.6	38.30
LED	2 L/3	Fatima	45.5 (39–52)	668.0	30.3	45.50

For the 2 L/3 treatment, fertile tillers, seed number, and seed mass are given per pot (three plants). All 24 pots are included; the mean TGW for the HP S 2 L/3 treatment of cv. Grace includes the early-harvested outlier pot (27.3 g) and is 42.5 g if that pot is excluded.

### 3.3 Lamp-light use efficiency

**Figure 2:**
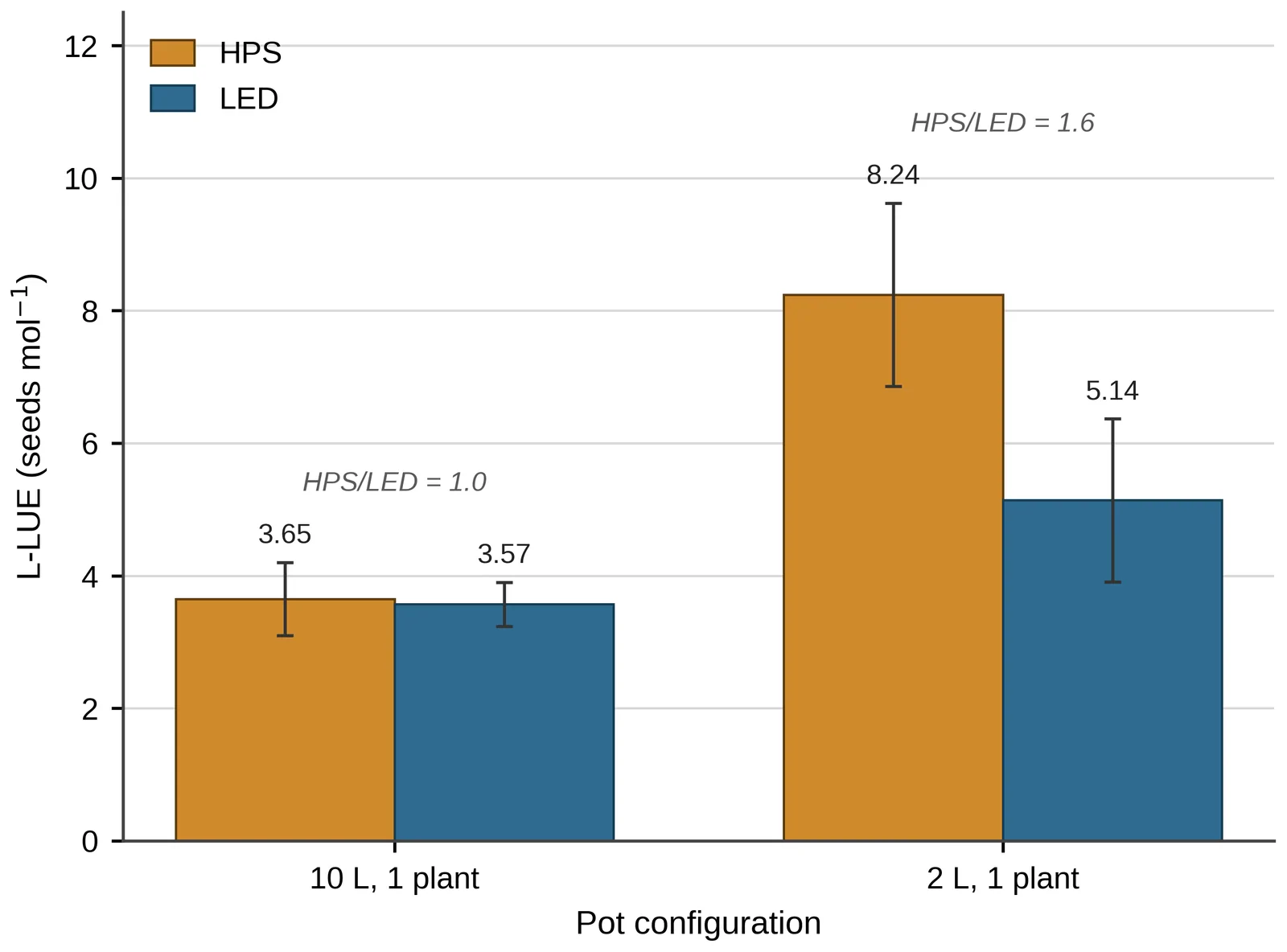
Lamp-light use efficiency (L-LUE) for the one-plant pot configurations under HP S and LED lighting. Values are seeds per mole of lamp-supplied photons (mean ± SD of the per-pot values, *n* = 4), calculated with daily light integrals of 37.2 mol m^−2^ day^−1^ (HP S) and 68.0 mol m^−2^ day^−1^ (LED). HP S matched or exceeded LED efficiency; the higher absolute yield under LED reflects a greater photon dose rather than a spectral advantage. Values for all six treatments are given in **Supplementary Table S4**.

The two systems differed roughly twofold in lamp-only DLI (~37 versus ~68 mol m^−2^ day^−1^). When yield was normalized per mole of lamp-supplied photons, HP S matched or exceeded LED efficiency at the same pot size (**Figure 2**). In 2 L pots, HP S was 1.6-fold more efficient (8.24 versus 5.14 seeds mol^−1^); in 10 L pots, efficiencies were nearly identical (3.65 versus 3.57 seeds mol^−1^). Hence, the higher absolute yield under LED reflects its larger photon dose, not a spectral advantage. Across all three pot configurations, the HP S treatment was at least as efficient per lamp-supplied photon as the LED treatment, with efficiency ratios of 1.0, 1.6, and 2.1 for the 10 L, 2 L single-plant, and 2 L three-plant configurations, respectively (**Supplementary Table S4**). The highest absolute value (9.64 seeds mol^−1^, HP S with three plants per 2 L pot) is partly attributable to the shorter growing period of that treatment (73–92 against 100 days under LED) rather than to spectral composition alone.

## 4 Discussion

Our results confirm that intensive off-season multiplication can close the gap between SB-accelerated line development and field-scale yield testing. Exceptional tillering and seed set (up to 105 fertile tillers, SMR up to 1,560) were achieved with both HP S and LED across two genetically distinct cultivars, demonstrating that the protocol does not depend on sophisticated spectrum tuning. Reproducibility across two cultivars of independent origin is grounds for testing the approach more widely rather than evidence that it is universal; a trial across a wider set of genotypes in at least two seasons is the necessary next step.

The high productivity arises from isolated plant growth (no neighbor shading, high red:far-red ratio promoting tiller production), high DLI (37–68 mol m^−2^ day^−1^), and ample root volume with correspondingly greater nutrient and water supply. Root-derived cytokinins promote tiller-bud outgrowth, whereas nutrient limitation in smaller pots raises strigolactones that suppress it (Umehara et al., 2010; Dun et al., 2012). A low red-to-far-red ratio suppresses tiller appearance in barley (Skinner and Simmons, 1993; Davis and Simmons, 1994) as part of the shade-avoidance syndrome (Casal, 2013); the lower far-red content of the LED spectrum may therefore have contributed to its higher tillering. The ~10-day-earlier heading under HP S is consistent with the accelerating effect of far-red light on photoperiodic floral induction in barley (Deitzer et al., 1979). However, because the systems also differed in photon flux, spectral and dose effects cannot be fully separated. A dedicated experiment with equalized DLI is needed. Such an experiment would use a single room without access to daylight and both types of luminaire dimmed to identical photon flux density and identical DLI, in a two-spectra × two-dose level arrangement. Until it has been performed, any conclusion about the role of spectral composition remains provisional. The higher L-LUE in 2 L pots (**Figure 2**) likely reflects a shift in partitioning: when root volume restricts vegetative growth, more assimilates are directed to grain. Per lamp-supplied photon, HP S matched or exceeded LED efficiency, reinforcing that the higher absolute LED yields are dose-driven. Comparable multiplication was therefore obtained under both of the lighting systems tested, so within this range of configurations lamp choice is a matter of local availability and economics.

**Figure 3:**
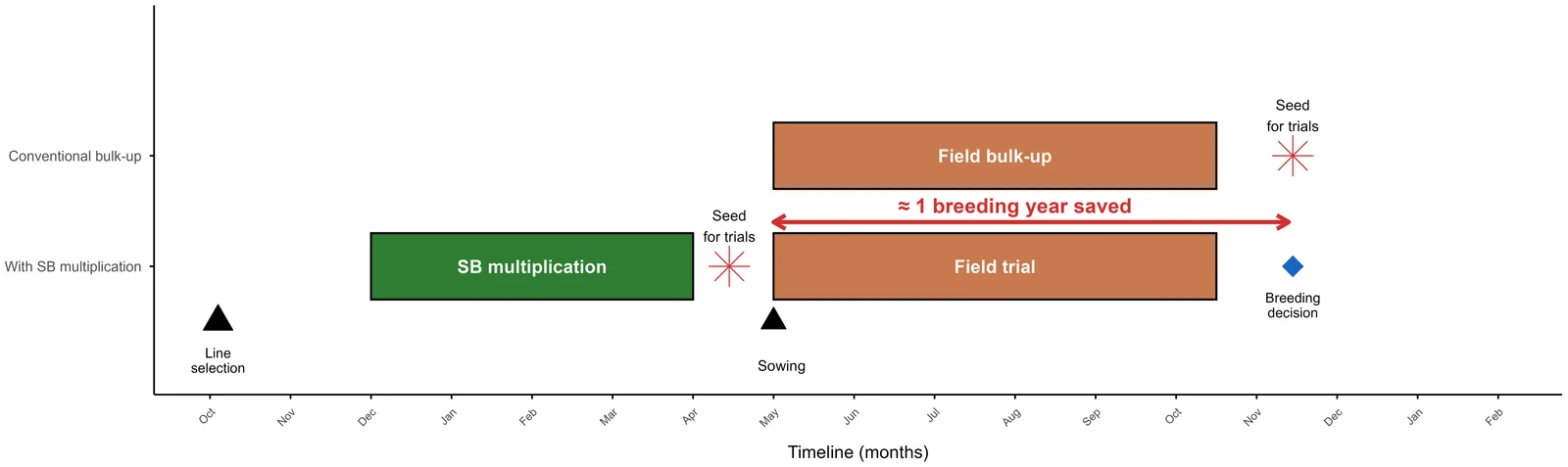
Off-season multiplication in the breeding calendar. Top track (terracotta): conventional field bulk-up delays trial sowing by 1 year. Bottom track (green + terracotta): off-season multiplication (December–April) provides seed ready for spring sowing. The red arrow indicates ≈1 breeding year saved.

In practice, breeders can select pot size according to their limiting resource: 10 L pots maximize seed per plant when starting seed is scarce; 2 L pots maximize seed per unit chamber area (**Table 2**). Because TGW was the only trait affected by cultivar, the size of a multiplication cycle should be planned from seed number rather than from seed mass, which varies with genotype. Because adding three plants per 2 L pot did not improve per-pot output, one plant per pot is recommended. This contrasts with the classic use of SB for single-seed descent, where dense planting is justified (Ghosh et al., 2018). **Figure 3** illustrates how off-season multiplication compresses the breeding calendar: a line selected in autumn is multiplied under a controlled environment (December–April) and sown directly into field trials the following spring, bypassing a field multiplication season. A competitive variety trial for barley typically requires ~30,000–80,000 seeds; at the best per-area yield observed (~24,000 seeds m^−2^), this quantity can be produced in ~1.3–3.3 m^2^ of chamber space. Earlier-stage trials need proportionally less area. Speed breeding can be used in two complementary modes: rapid generation advance, in which immature ears are harvested to cycle generations as fast as possible and fix a line, and intensive off-season multiplication, in which plants are taken to full maturity for maximum seed output (the approach studied here). If the pipeline is designed to switch from the first mode to the second at the right moment—using rapid generation advance to reach a near-homozygous line in fewer calendar years, then switching to intensive multiplication to bulk that line off-season to trial scale-up to two breeding years can be saved: roughly one from faster generation advance and one from bypassing the field bulk-up seasons and entering competitive trials directly. The high multiplication ratio also accelerates the build-up of grain mass, potentially saving an additional season when larger seed quantities are required.

We acknowledge several limitations. Replication was two pots per cell; although each factor level is represented by 8 to 12 pots, the design could detect only differences larger than approximately 175 seeds for a main effect, so effect sizes (*η*²) and confidence intervals guide interpretation, and non-significant effects are reported as differences that were not detected rather than as differences shown to be absent. Only two cultivars, one site, and one winter season were tested, so the study is a proof of concept rather than a general demonstration. The lighting comparison confounds spectrum, photon flux, and daylight exposure: the two systems are treated as integral technological configurations, and conclusions about spectral composition remain provisional until an experiment with equalized DLI has been performed. The pot-size effect confounds rooting volume with total nutrient and water supply, because fertilizer was applied at a constant rate per liter of substrate. L-LUE estimates assume full light interception and are therefore upper bounds, although the ratio between the two systems at a given pot size is robust. Finally, the SMR is expressed per seed sown and is not directly comparable with the field multiplication coefficient, which is a mass ratio per unit area; the appropriate field comparator is the per-plant seed number. None of these limitations affects the main conclusion that field-trial quantities of seed can be produced in a single off-season cycle under either of the lighting systems tested.

In summary, intensive off-season multiplication is a simple way to bulk SB-derived lines to field-trial quantities within a single winter cycle, and comparable output was obtained under both of the lighting systems tested. Key practical recommendations are as follows: (1) use 10 L pots when seed is limiting and 2 L pots when chamber area is limiting; (2) grow one plant per pot; (3) plan the size of a cycle from seed number rather than seed mass; and (4) follow rapid generation advance with a single off-season multiplication cycle, which can save up to two breeding years provided that the pipeline is designed to switch from rapid generation advance to maximum-productivity multiplication at the appropriate point. The approach requires no equipment beyond a controlled-environment room with an extended photoperiod and either high-pressure sodium or LED lighting.

## Data Availability

The raw data supporting the conclusions of this article will be made available by the authors, without undue reservation.
