# Supplementary material for: Off-season seed multiplication in spring barley: a scale-up step for speed breeding
Source: Front Plant Sci. 2026 Aug 20;17:1912336. doi: 10.3389/fpls.2026.1912336 (PMC13538881; doi:10.3389/fpls.2026.1912336)
Supplement: Supplementary file 1 [file DataSheet1.docx]

Supplementary Material

**Supplementary Methods.** Details of irrigation, pest control, fertiliser application and statistical analysis.

Plants were watered daily, avoiding both waterlogging and drying out of the substrate. Watering frequency was reduced when plants reached the soft-dough stage. Integrated pest control was applied throughout the experiment, including regular monitoring and the application of standard control measures as needed. Statistical analysis. The experiment was a balanced factorial with 2 lighting systems × 3 pot configurations × 2 cultivars × 2 replicates (24 pots). All 24 pots were retained in every model, so the design remained fully balanced and sequential (Type I), Type II and Type III sums of squares are identical; no contrast recoding was therefore required. The full model (Supplementary Table S2) included all main effects and all interactions. No interaction involving cultivar approached significance (p ≥ 0.09), and these terms were dropped to give the reduced model reported in Table 1 (residual df = 17). Because the two models differ in residual degrees of freedom (12 and 17 respectively), their F values are not directly comparable, whereas the effect sizes are.

One pot (HPS, 2 L with three plants, cv. Grace, replicate 1) had a thousand-grain weight of 27.3 g, identified as an outlier by Grubbs’ test (p < 0.05) and attributable to incomplete grain filling in a sample harvested at 73 days. It was retained in the main analysis; Supplementary Table S3 reports the same reduced model fitted after excluding this pot (Type II sums of squares, residual df = 16). The two analyses lead to the same conclusions.

The sensitivity of the design was expressed as the minimum detectable difference at 80 % power and α = 0.05, calculated from the residual mean square of the seed-number model (MSE = 20,708; residual standard deviation 144 seeds per pot). Retrospective power computed from the observed effects was not used, because it is a monotone function of the achieved p-value. Residual diagnostics comprised the Shapiro–Wilk test and visual inspection of residual plots.

Lamp-light use efficiency (L-LUE) was calculated for each pot as seeds (or grams) per mole of lamp-supplied photons, the photon supply being the daily light integral × days to maturity × pot footprint. Daily light integrals were 37.2 mol m⁻² d⁻¹ for HPS (470 µmol m⁻² s⁻¹ over 22 h) and 68.0 mol m⁻² d⁻¹ for LED (858 µmol m⁻² s⁻¹ over 22 h). Treatment values are reported as means of the per-pot values.

**Supplementary Table S1.** Characteristics of the two studied spring barley cultivars.

| **Trait** | **Grace** | **Fatima** |
| --- | --- | --- |
| Botanical variety | *nutans* (two-rowed) | *nutans* (two-rowed) |
| Maturity group | mid-season | mid-season |
| Field growing period, days | 70–85 | 74–92 |
| End use | malting | malting |
| Thousand-grain weight, g | 39–50 | 42–52 |
| Pedigree | (Xanadu × Simba) × Marnie | Tam Tam × Sunshine |
| Originator | Ackermann Saatzucht (Germany) | Secobra Recherches (France) |

**Supplementary Table S2.** Full three-way analysis of variance for fertile tillering, seed number, seed mass and thousand-grain weight (TGW), including all interactions. All 24 pots were included; residual df = 12. F values are given with their significance, and effect sizes (η², % of total variation) in parentheses. No interaction involving cultivar approached significance (smallest p = 0.088, Pot × Cultivar for TGW); these terms were dropped to give the reduced model reported in Table 1. Because the reduced model has more residual degrees of freedom (17), its F values are larger than those shown here, whereas the η² values are directly comparable. ***p < 0.001; **p < 0.01; *p < 0.05; ns, not significant.

| **Source of variation** | **Fertile tillers** | **Seed number** | **Seed mass** | **TGW** |
| --- | --- | --- | --- | --- |
| Lighting | 48.33 *** (35.8) | 20.79 *** (14.4) | 9.28 * (9.8) | 0.78 ns (2.4) |
| Pot configuration | 26.89 *** (39.8) | 43.04 *** (59.8) | 28.02 *** (59.4) | 1.94 ns (11.9) |
| Cultivar | 0.02 ns (0.0) | 0.10 ns (0.1) | 0.86 ns (0.9) | 8.21 * (25.0) |
| Lighting × Pot | 10.00 ** (14.8) | 10.91 ** (15.2) | 5.42 * (11.5) | 0.46 ns (2.8) |
| Lighting × Cultivar | 0.14 ns (0.1) | 0.01 ns (0.0) | 0.25 ns (0.3) | 0.95 ns (2.9) |
| Pot × Cultivar | 0.29 ns (0.4) | 1.37 ns (1.9) | 2.40 ns (5.1) | 2.99 ns (18.2) |
| Lighting × Pot × Cultivar | 0.12 ns (0.2) | 0.20 ns (0.3) | 0.11 ns (0.2) | 0.04 ns (0.2) |
| Residual | — (8.9) | — (8.3) | — (12.7) | — (36.6) |

**Supplementary Table S3.** Sensitivity analysis: the reduced model (Lighting + Pot + Cultivar + Lighting × Pot) fitted after excluding the pot with an outlying thousand-grain weight (HPS, 2 L with three plants, cv. Grace, replicate 1; TGW 27.3 g). The design is unbalanced once this pot is removed, so Type II sums of squares are reported; residual df = 16. Effect sizes (η², % of total variation) are given in parentheses. The pattern of significance is identical to that of the main analysis based on all 24 pots (Table 1) for every trait, including the effect of cultivar on TGW (p = 0.022 here against p = 0.015 in the main analysis). ***p < 0.001; **p < 0.01; *p < 0.05; ns, not significant.

| Source of variation | Fertile tillers | Seed number | Seed mass | TGW |
| --- | --- | --- | --- | --- |
| Lighting | 59.44 *** (35.6) | 23.54 *** (15.3) | 8.30 * (9.5) | 3.30 ns (12.0) |
| Pot configuration | 33.42 *** (40.1) | 46.35 *** (60.1) | 25.80 *** (59.2) | 0.85 ns (6.2) |
| Cultivar | 0.01 ns (0.0) | 0.14 ns (0.1) | 0.52 ns (0.6) | 6.44 * (23.5) |
| Lighting × Pot | 12.30 *** (14.7) | 10.87 ** (14.1) | 5.40 * (12.4) | 0.00 ns (0.0) |
| Residual | — (9.6) | — (10.4) | — (18.3) | — (58.3) |

**Supplementary Table S4**. Lamp-supplied daily light integral (DLI, mol m⁻² d⁻¹), photon supply and lamp-light use efficiency (L-LUE) for all six treatments. Photon supply is DLI × days to maturity × pot footprint; pot footprints were 0.065 m² (10 L) and 0.0196 m² (2 L). L-LUE values are means ± SD of the per-pot values (n = 4). Across all three pot configurations, HPS was at least as efficient per lamp-supplied photon as LED, the efficiency ratios being 1.0, 1.6 and 2.1. The high value for HPS 2 L / 3 partly reflects the shorter growing period of that treatment rather than spectral composition alone. The LED treatment additionally received an estimated 1–3 mol m⁻² d⁻¹ of daylight, which is not included here and which therefore makes the comparison conservative.

| Treatment | DLI | Days | Photons, mol | L-LUE, seeds mol⁻¹ | L-LUE, g mol⁻¹ |
| --- | --- | --- | --- | --- | --- |
| HPS, 10 L / 1 | 37.2 | 92–105 | 240.1 | 3.65 ± 0.55 | 0.173 ± 0.023 |
| HPS, 2 L / 1 | 37.2 | 92 | 67.1 | 8.24 ± 1.38 | 0.383 ± 0.104 |
| HPS, 2 L / 3 | 37.2 | 73–92 | 60.2 | 9.64 ± 1.15 | 0.387 ± 0.058 |
| LED, 10 L / 1 | 68.0 | 92–105 | 435.1 | 3.57 ± 0.33 | 0.160 ± 0.031 |
| LED, 2 L / 1 | 68.0 | 100 | 133.2 | 5.14 ± 1.23 | 0.219 ± 0.051 |
| LED, 2 L / 3 | 68.0 | 100 | 133.2 | 4.53 ± 0.88 | 0.191 ± 0.052 |

**Supplementary Table S5.** Per-pot raw data for all 24 experimental units. L-LUE, lamp-light use efficiency (seeds per mole of lamp-supplied photons). † This pot had an outlying thousand-grain weight (27.3 g; Grubbs’ test, p < 0.05) attributable to harvest at 73 days with incomplete grain filling. It is retained in the main analysis; see Supplementary Table S3 for the sensitivity analysis in which it is excluded. Its high L-LUE follows from the short growing period, which reduces the photon supply in the denominator.

| **System** | **Pot / pl.** | **Cultivar** | **Rep** | **Days** | **Fertile tillers** | **Total tillers** | **Seed number** | **Seed mass, g** | **TGW, g** | **L-LUE, seeds mol⁻¹** |
| --- | --- | --- | --- | --- | --- | --- | --- | --- | --- | --- |
| HPS | 10 L/1 | Grace | 1 | 92 | 50 | 55 | 955 | 42.94 | 44.96 | 4.29 |
| HPS | 10 L/1 | Grace | 2 | 100 | 46 | 87 | 885 | 44.00 | 49.66 | 3.66 |
| HPS | 10 L/1 | Fatima | 1 | 105 | 50 | 108 | 748 | 35.70 | 47.75 | 2.94 |
| HPS | 10 L/1 | Fatima | 2 | 100 | 36 | 41 | 895 | 42.30 | 47.30 | 3.70 |
| HPS | 2 L/1 | Grace | 1 | 92 | 28 | 28 | 521 | 20.40 | 39.21 | 7.76 |
| HPS | 2 L/1 | Grace | 2 | 92 | 26 | 33 | 443 | 19.10 | 43.14 | 6.60 |
| HPS | 2 L/1 | Fatima | 1 | 92 | 38 | 51 | 658 | 32.80 | 49.78 | 9.80 |
| HPS | 2 L/1 | Fatima | 2 | 92 | 33 | 43 | 591 | 30.50 | 51.66 | 8.80 |
| HPS | 2 L/3 | Grace | 1 | 73 | 29 | 32 | 598 | 16.30 | 27.26 † | 11.23 |
| HPS | 2 L / 3 | Grace | 2 | 73 | 32 | 35 | 519 | 22.00 | 42.47 | 9.74 |
| HPS | 2 L / 3 | Fatima | 1 | 92 | 32 | 56 | 601 | 29.50 | 49.10 | 8.95 |
| HPS | 2 L/3 | Fatima | 2 | 92 | 36 | 51 | 580 | 26.20 | 45.16 | 8.64 |
| LED | 10 L/1 | Grace | 1 | 92 | 87 | 161 | 1457 | 57.00 | 39.14 | 3.59 |
| LED | 10 L/1 | Grace | 2 | 105 | 125 | 168 | 1819 | 95.20 | 52.34 | 3.92 |
| LED | 10 L/1 | Fatima | 1 | 105 | 115 | 199 | 1697 | 71.40 | 42.07 | 3.66 |
| LED | 10 L/1 | Fatima | 2 | 92 | 92 | 146 | 1273 | 57.10 | 44.83 | 3.13 |
| LED | 2 L/1 | Grace | 1 | 100 | 46 | 58 | 515 | 21.90 | 42.52 | 3.87 |
| LED | 2 L/1 | Grace | 2 | 100 | 57 | 81 | 792 | 29.40 | 37.16 | 5.95 |
| LED | 2 L/1 | Fatima | 1 | 100 | 45 | 62 | 578 | 27.10 | 46.89 | 4.34 |
| LED | 2 L/1 | Fatima | 2 | 100 | 59 | 86 | 856 | 38.20 | 44.63 | 6.43 |
| LED | 2 L/3 | Grace | 1 | 100 | 53 | 83 | 565 | 22.90 | 40.57 | 4.24 |
| LED | 2 L/3 | Grace | 2 | 100 | 40 | 84 | 512 | 18.40 | 36.02 | 3.84 |
| LED | 2 L/3 | Fatima | 1 | 100 | 39 | 70 | 560 | 25.90 | 46.21 | 4.20 |
| LED | 2 L/3 | Fatima | 2 | 100 | 52 | 96 | 776 | 34.70 | 44.80 | 5.83 |

**Supplementary Figure S1.** Seed number per plant across treatments (boxplots).
